# Novel Tannic Acid-Modified Cobalt-Based Metal–Organic Framework: Synthesis, Characterization, and Antimicrobial Activity

**DOI:** 10.1021/acsomega.3c09169

**Published:** 2024-04-16

**Authors:** Elif Ant Bursalı

**Affiliations:** Department of Chemistry, Dokuz Eylul University, Tınaztepe, Izmir 35390, Turkiye

## Abstract

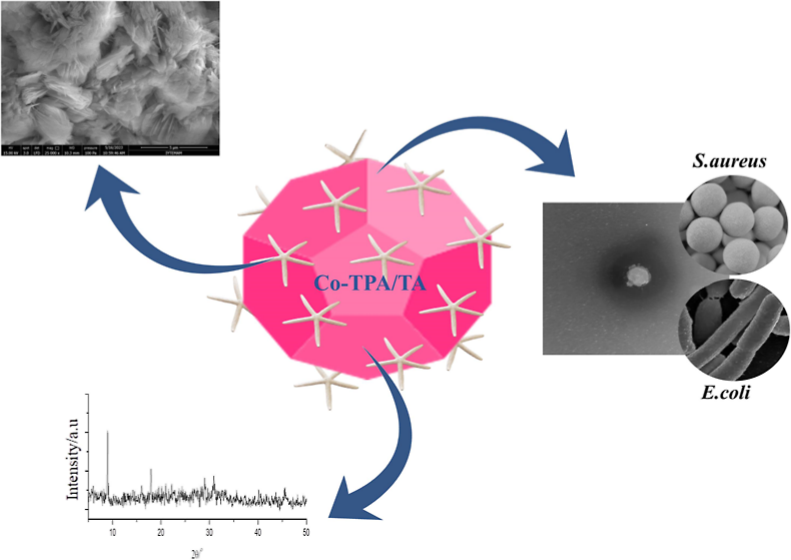

Metal–organic frameworks (MOFs) are a class of hybrid inorganic–organic materials with typical porous structures and a unique morphology. Due to their diversity, they are extensively used in a wide range of applications such as environmental, catalysis, biomedicine, etc. In this study, a novel cobalt-based MOF modified with tannic acid (Co-TPA/TA) (TPA: terephthalic acid; TA: tannic acid) as a promising material for antimicrobial agents was synthesized and characterized by X-ray diffraction, Fourier transform infrared spectroscopy, scanning electron microscopy with energy-dispersive X-ray spectroscopy, X-ray photoelectron spectroscopy, inductively coupled plasma-optical emission spectrometry, and thermogravimetric analysis and compared with an as-synthesized cobalt-based framework. Co-TPA/TA demonstrated good antimicrobial efficiency under optimum conditions against yeast *Candida albicans* ATCC 10231, Gram-negative *Escherichia coli* ATCC 8739, and Gram-positive *Staphylococcus aureus* ATCC 6538 with an inhibition zone ranging from 14 to 20 mm. Reduced ATP levels, generation of reactive oxygen species, membrane damage from cobalt ion release, and development of an alkaline microenvironment could all be contributing factors to the possible antimicrobial pathways. The novel framework can be obtained using simple, affordable, and easily accessible commercial ligands and is considered to have the potential to be used as an antimicrobial material in the future.

## 1 Introduction

The increase in the number of resistant pathogens against antibiotics and infectious diseases has caused many health problems for humans. As a result, researchers are very interested in newly developed antimicrobial materials for sterilizing and suppressing bacterial development. Several materials have been utilized for this purpose, including organic^1^ or natural materials,^2^ photocatalytic materials,^3,4^ and polymer composites;^5,6^ however, most of them have some shortcomings. Metal–organic frameworks (MOFs) are alternative antimicrobial materials for various medical applications due to their high loading and controlled release capacities, easy functionalization, and strong interactions with bacterial membranes, as well as excellent biodegradability and biocompatibility.^7−10^

MOFs are an important type of hybrid organic–inorganic porous crystalline material with unique morphologies. In more than 20 years since the first porous MOF was initially introduced, more than 2000 MOF topologies have been defined. They are two- or three-dimensional structures obtained by bonding of ionic metals or metal clusters with organic ligands, in which metal ions are coordination centers of the structure, and organic ligands are linkers between these centers. MOFs have recently gained attention because they are highly suited for a range of applications, including chromatographic separation,^11,12^ gas storage,^13,14^ molecular sieving,^15,16^ chemical/biosensors,^17,18^ medical imaging,^19,20^ heterogeneous catalysis,^21,22^ and drug release,^23,24^ etc., due to their vast surface areas and predictable and controllable pore diameters. These adjustable properties and structures of MOFs depend on the central metal ion, ligand structure, metal–ligand ratio, type of solvent, pH, and temperature. Several metals and various multidentate ligands have been used to build MOFs with diverse structures and topology. Among these metals and ligands, cobalt is preferred as an inexpensive element with high antimicrobial activity and terephthalic acid; an aromatic carboxylic acid that has equally spaced carboxylate groups is frequently used because of the rigidity of the phenyl skeleton and it can create versatile types of coordination.^25−28^

Besides, incorporating an adjustable surface on the MOF to control the surface properties may be required. Recently, the provision of a metal-phenolic coordination coating on the MOF surface has gained particular interest in terms of biocompatibility and improving microbial activity. Tannic acid (TA) is a macromolecule with a large number of hydroxyl groups and is easily gained from plants; therefore, it is relatively cheaper than the other polyphenols. TA is known for its favorable properties such as biocompatibility, antioxidant activity, and antimicrobial properties.^29−31^

This study aims to create a new MOF based on cobalt that has been modified with TA (Co-TPA/TA) to investigate the antimicrobial activity of this framework and to compare it with the as-synthesized Co-based MOF (Co-TPA). During the synthesis of MOFs, terephthalic acid (TPA) was used as a commercial organic ligand. The MOFs were characterized by X-ray diffraction (XRD), Fourier transform infrared spectroscopy (FTIR), scanning electron microscopy with energy-dispersive X-ray spectroscopy (SEM-EDX), X-ray photoelectron spectroscopy (XPS), inductively coupled plasma—optical emission spectrometry (ICP-OES), and thermogravimetric (TGA) analysis. The antimicrobial activity was investigated through a modified agar well-diffusion method. *Staphylococcus aureus* and *Escherichia coli* as Gram-positive and Gram-negative bacteria, respectively, and *Candida albicans* as yeast were used as different types of microorganisms. The antimicrobial efficiency of the MOFs was evaluated in comparison with that of the standard antibiotic, amoxicillin.

## 2 Experimental Section

### 2.1 Chemicals

Cobalt nitrate hexahydrate (Co(NO_3_)_2_·6H_2_O), terephthalic acid (benzene-1,4,-dicarboxylic acid, TPA), tannic acid (TA), *N*,*N*-dimethylformamide (DMF), methanol, and ethanol were purchased from Fluka, Sigma-Aldrich, or Merck and were of analytical reagent grade. Ultrapure water was used during the experiments. Tryptic Soy Agar (Merck) and Sabouraud Dextrose Agar (Merck) were used in antimicrobial activity tests.

### 2.2 Instrumentation

FTIR spectra were obtained on a Perkin–Elmer Spectrum BX-II Model FTIR spectrophotometer from 4000 to 400 cm^–1^ at a 4 cm^–1^ resolution using KBr pellets. XRD results were provided by operating with CuKα (λ = 1.54 Å) radiation at a 2θ range from 2 to 50° using a Thermo Scientific ARL X’TRA diffractometer. The morphologies and compositions of the MOFs were examined using an FEI Quanta 250 FEG SEM scanning electron microscope equipped with an energy-dispersive X-ray spectrometer operating at a 15 kV acceleration voltage. The MOFs were coated with a thin gold layer before imaging. XPS analyses of the MOFs were conducted by a Thermo-Scientific K-Alpha instrument with a monochromatic Al-Kα source (1486.7 eV) and a beam size of 400 nm diameter. The XPS survey spectra were obtained from a single point, with 15 scans between 10 and 1350 eV. ICP-OES measurements were performed on a Thermo Scientific iCAP 600 Series instrument. The solid samples were dissolved by microwave-assisted acid digestion (EPA 3051 method). The quantitative determination of Co was achieved by extrapolating from a five-point calibration curve. The measurements were conducted in triplicate, and the results were given as averages. TGA analysis using PerkinElmer STA 6000 thermogravimetry/differential thermal analyzer was performed from 35 to 600 °C under a nitrogen atmosphere with a heating rate of 10 °C/min in porcelain pans.

### 2.3 Synthesis of Co-TPA and Co-TPA/TA

Co-TPA was synthesized by revising similar synthesis methods seen in the literature.^32−34^ 0.291 g of Co(NO_3_)_2_·6H_2_O in 2.5 mL of ultrapure water and 0.166 g of TPA in 12.5 mL of DMF were mixed and, after 30 min of sonication, were refluxed at 100 °C for 48 h under a nitrogen atmosphere. The obtained solid was washed several times with methanol and dried at 80 °C overnight.

**Figure 1:**
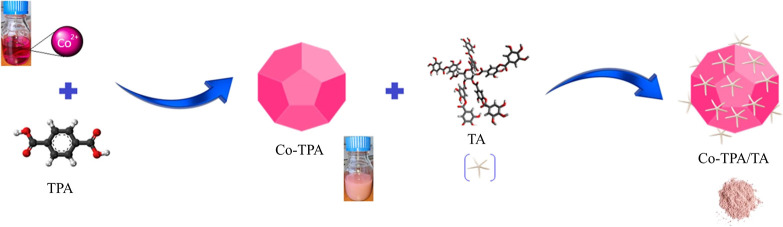
Schematic illustration of the synthesis of Co-TPA and Co-TPA/TA.

The modification of Co-TPA with TA was performed similarly to the procedure described by Ge et al.^35^ 0.2 g of Co-TPA was dispersed in 4 mL of ethanol, and it was sonicated for 15 min to get a homogeneous dispersion system. While 0.50 g of TA was dispersed in 96 mL of ultrapure water to form a solution. Then the TA solution was slowly poured into the solution of Co-TPA, and after 30 min of sonication, solid powder, denoted as Co-TPA/TA, was collected by centrifugation, washed with methanol and water several times, and dried at 80 °C overnight in a vacuum oven. The reaction pathway for the synthesis is illustrated in Figure 1.

### 2.4 Antimicrobial Activities

Antimicrobial activities of Co-TPA, Co-TPA/TA, and a standard antibiotic (amoxicillin) were tested against three strains of microorganisms, including Gram-positive (*S. aureus* ATCC 6538) and Gram-negative (*E. coli* ATCC 8739) bacteria and yeast (*C. albicans* ATCC 10231) using a modified agar well-diffusion method described by Paik and Glatz.^36^ Bacterial cultures were incubated on Tryptic Soy Agar medium for 18–24 h at 37 °C, and yeast culture was incubated at 30 °C for 48 h after inoculation on Sabouraud Dextrose Agar medium. Before inoculation, the plates were dried at 35 °C for 40 min in an incubator. Three to five freshly grown colonies of bacterial strains and yeast culture were inoculated into 50 mL of Tryptic Soy broth medium and Sabouraud Dextrose broth in a shaking water bath for 4 to 6 h until a turbidity of 0.5 McFarland (1 × 10^8^ CFU/mL) was reached. The final inocula were adjusted to 5 × 10^5^ CFU/mL using a spectrophotometer.^37^ Six-millimeter diameter wells were cut from the agar by using a sterile cork-borer, and samples (Co-TPA and Co-TPA/TA) were transferred to the wells and allowed to diffuse at RT for a maximum of 2 h. Each substance was utilized in a powder form, with 1 mg of each being applied directly to the inoculated agar plate. Antimicrobial activity was evaluated by the zone of inhibition of the growth of the test microorganism. The tests were carried out in triplicate. Wells which were filled with noninoculated medium served as controls. Standard antibacterial disks of amoxicillin (A/30) were individually used as positive control. An inhibition zone ruler was used to measure the diameters of the inhibition zones in mm.

## 3 Results and Discussion

### 3.1 Characterization Studies

**Figure 2:**
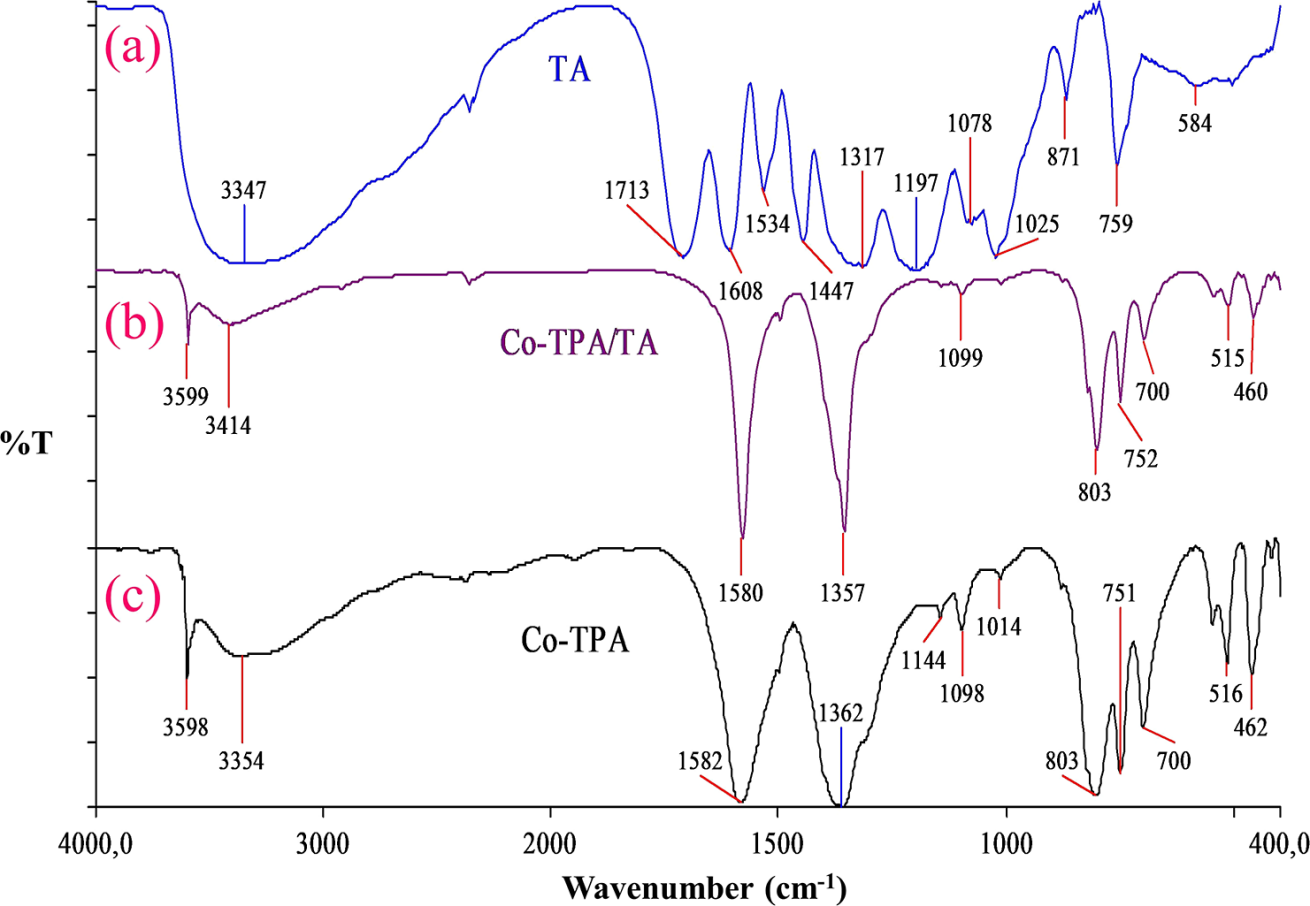
FTIR spectra of Co-TPA (a), Co-TPA/TA (b), and TA (c).

FTIR spectra of TA, Co-TPA, and Co-TPA/TA are shown in Figure 2a–c. In all spectra, the broad bands centered at around 3450–3300 cm^–1^ were typical OH bond vibrations of water molecules. In the spectrum of TA (Figure 2a), characteristics of carbonyl group C=O stretching were seen at 1713 cm^–1^. The bands of aromatic C–C bonds at 1608 and 1534 cm^–1^ and C–C deformation vibrations at 1447 and 1317 cm^–1^ were attributed to the phenolic groups. The band observed at 1197 cm^–1^ was attributed to the aromatic C–O deformation. The bands in the region of 1100–1000 cm^–1^ were due to C–O stretching and C–H deformations while the C–H deformation vibrations of the benzene ring were also seen below 900 cm^–1^.

The FTIR spectra of the as-synthesized Co-TPA (Figure 2c) demonstrated strong COO– asymmetric and symmetric stretching bands, which were attributed to the carboxylate group of the TPA linker at around 1582 and 1360 cm^–1^, respectively. The expected strong stretching vibration of carboxylic acid of TPA (at around 1685 cm^–1^) was not observed in the spectrum, but it was separated into two bands, confirming that the COO– group was in coordination with metallic cobalt.^27,38^

The characteristic bands observed between 800 and 1150 cm^–1^ were due to aromatic C–H bonds of TPA.^28^ The bands at around 1098 and 800–700 cm^–1^ were related to C–O–Co and Co–O stretching vibrations, respectively, indicating good coordination of carboxylic acid group of TPA and cobalt ions.^39−41^ The spectrum of Co-TPA/TA (Figure 2b) appears to be quite similar to the spectrum of Co-TPA (Figure 2c) demonstrating the same bond features with slight shifts (1–5 cm^–1^) in the frequencies of peaks except for the OH group. The noticeable decrease in the intensities of the bands in Co-TPA/TA could suggest the successful coordination of phenolic −OH groups in benzene rings of TA with cobalt ion. Also, some of the characteristic bands of TA (Figure 2a) did not appear in the spectrum of Co-TPA/TA due to the high intensity of the bands of Co-TPA.

**Figure 3:**
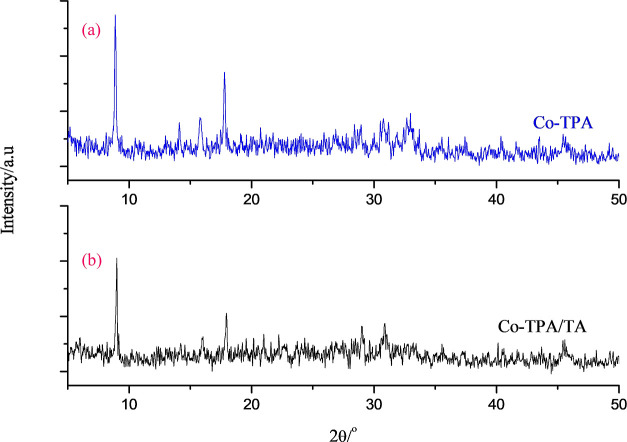
XRD patterns of Co-TPA (a) and Co-TPA/TA (b).

XRD analysis results of Co-TPA and Co-TPA/TA are given in Figure 3. In the XRD pattern of Co-TPA (Figure 3a), the diffraction peaks at 8.9, 14.1, 15.8, 17.8, 28.9, 30.8, 32.9, 40.3, and 45.4° correspond to (002), (121), (131), (141), (301), (133), (153), (333), and (264), respectively. In the XRD pattern of Co-TPA/TA (Figure 3b), peaks belonging to Co-TPA were observed, except for 14.1 and 32.9° with small shifts (1–2°). These peaks revealed that the prepared materials are crystalline in nature, and the crystal structure of Co-TPA could be suggested as orthorhombic. The average crystallite sizes of Co-TPA and Co-TPA/TA were calculated to be ∼19 and ∼12 nm, respectively, from the XRD patterns using the Debye–Scherrer eq 1.
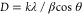
1where *D* is crystal size, *k* is the shape factor (0.9), θ is the Bragg angle, β is full width at half-maximum (fwhm) of the peak, and λ is the wavelength of X-ray. The peaks between 2θ = 17–35° in the patterns were attributed to the diffraction peaks of the organic ligand, TPA.^42^ The as-synthesized Co-TPA exhibited characteristic peaks of typical MOFs, and the values were in harmony with the reported ones of similar MOFs encountered in the literature.^27,28,32^ The expected metallic cobalt diffraction peaks originated from the cubic and hexagonal close pack forms at 2θ = 44 and 47°, respectively,^43^ were observed with very low intensities in the patterns of both Co-TPA and Co-TPA/TA, which showed that the cobalt ions were well-coordinated with the TPA ligand in both materials.^27,38^ TA insertion to the matrix slightly alters the basic structure of the MOF; consequently the intensities of the diffraction peaks of Co-TPA/TA were diminished and some of the peaks were a little bit shifted compared to Co-TPA, as seen from Figure 3a,b, demonstrating the relatively lower crystallinity of Co-TPA/TA. This result is consistent with the amorphous structure of the added polyphenol. Also, the diffraction peaks located at 2θ = 14.1 and 32.9° in the pattern of Co-TPA were not observed for Co-TPA/TA, indicating the effect of successful TA modification.

**Figure 4:**
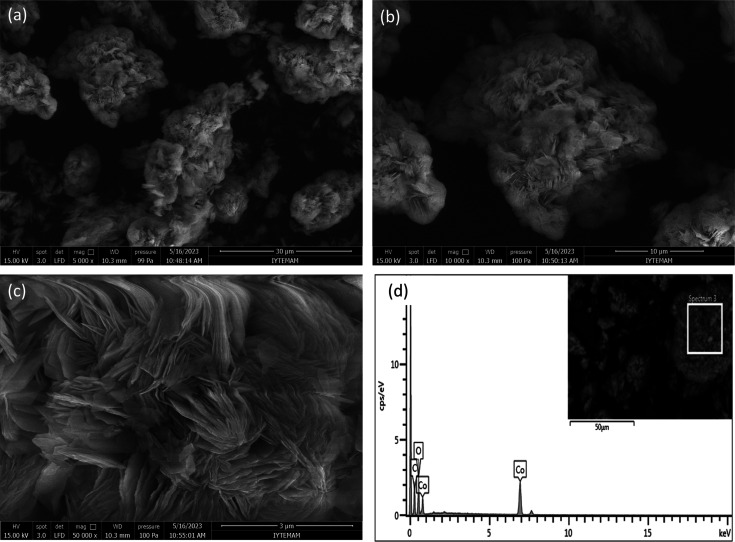
SEM images at 30, 10, and 3 μm magnifications (a–c) and EDX pattern (d) of Co-TPA.

**Figure 5:**
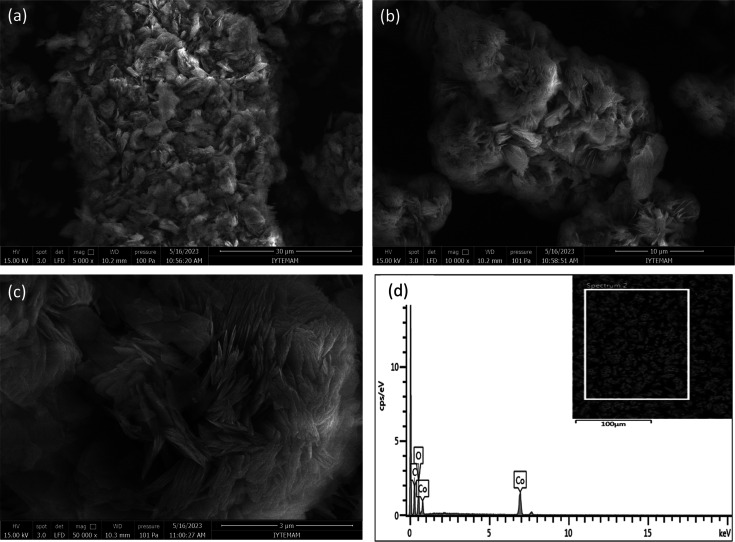
SEM images at 30, 10, and 3 μm magnifications (a–c) and EDX pattern (d) of Co-TPA/TA.

SEM studies revealed the morphologies, while EDX analysis identified the elemental distributions of Co-TPA and Co-TPA/TA, as shown in Figures 4 and 5, respectively. SEM images were obtained at 3, 10, and 30 μm magnifications. Figure 4a–c exhibits the morphology of the Co-TPA matrix; it can be easily seen that its crystalline structure was well-defined and ordered, being associated with a sheet-like appearance. By comparison, in Figure 5a–c, where the SEM images of Co-TPA/TA are presented, no obvious difference could be seen after the addition of TA. Its ordered structure seemed disturbed, resulting in blurred-like domains, suggesting that TA covered the Co-TPA.

Moreover, by EDX patterns (Figures 4d and 5d) the elemental composition of the matrices was established. It was presented that Co-TPA and Co-TPA/TA were primarily composed of Co, C, and O, as expected, and their contents are given in Table 1. These results confirmed the presence of Co within the studied matrices. With the successful introduction of polyphenol groups of TA into the matrix, the percentage of Co in Co-TPA/TA decreased slightly compared to Co-TPA due to the presence of C and O elements from TA.

**Table 1:** Results of EDX and ICP-OES Analyses of the Prepared Materials

	EDX (wt %)		ICP-OES (wt %)	
	Co-TPA	Co-TPA/TA	Co-TPA	Co-TPA/TA
Co	20.99	15.68	27.50	27.10
O	59.00	62.47		
C	20.01	21.85		

The composition of MOFs was further determined by ICP-OES, and the amounts of Co were found to be 27.50 and 27.10 wt % for CO-TPA and Co-TPA/TA, respectively. Compared to EDX (Table 1), higher metal contents were determined by ICP-OES on digested MOFs, considering that EDX focuses just on surface components, which was consistent with the expected result.

**Figure 6:**
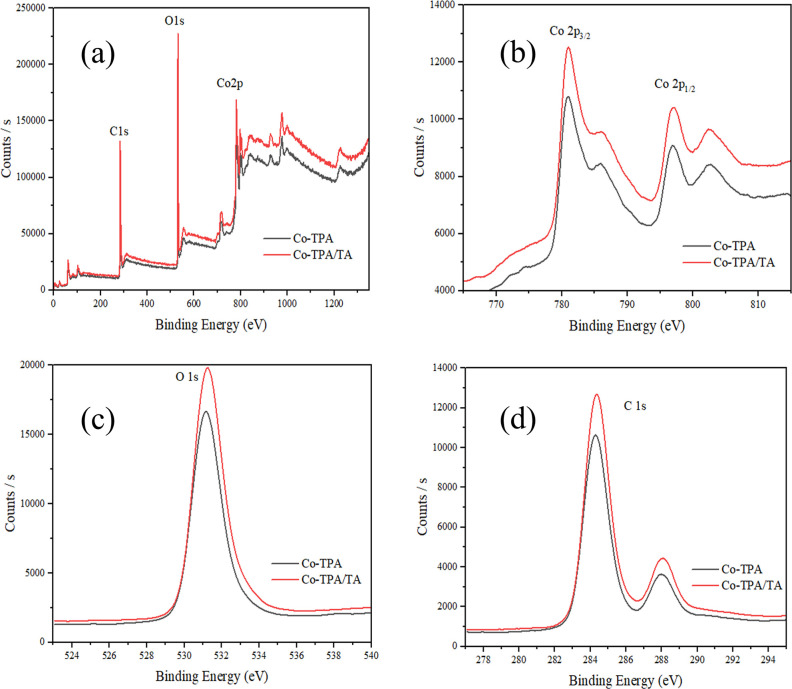
XPS survey (a) and expanded XPS spectra Co 2p (b), O 1s (c), and C 1s (d) of MOFs.

Elemental distribution of the MOFs was also evaluated by XPS measurements. The binding energies (eV) and atomic weight (%) values of available elements are given in Table 2 and the XPS spectra of the MOFs are represented in Figure 6. The XPS analysis results indicated the successful formation of the MOFs while supporting the EDX and ICP-OES results and were compatible with the literature.^44,45^ XPS survey spectra of Co-TPA and Co-TPA/TA both illustrated the presence of C, O, and Co elements with sharp peaks situated at binding energies of approximately 284 eV (C 1s), 531 eV (O 1s), and 781 eV (Co 2p) (Figure 6a). In the Co 2p XPS spectra of both MOFs (Figure 6b), two characteristic peaks observed at around 781 and 798 eV belong to cobalt nodes (Co 2p_3/2_ and Co 2p_1/2_) of Co^2+^, respectively, and these peaks had two distinct satellites (at ∼786 and ∼803 eV). Only one O 1s peak (Figure 6c) located at 531 eV was observed related to Co–O units of the synthesized MOFs, and there were no peaks observed for the oxygens of the TPA ligand (C–O and C=O) due to the similar coordination of the ligand to the metal center. Besides C 1s for both Co-TPA and Co-TPA/TA showed (Figure 6d) binding energies that can be assigned to C–C units at 284.28 and 284.35 eV and O–C=O units at 288.5 and 288.3 eV, respectively.^46,47^

**Table 2:** Binding Energy (eV) and Weight (%) Values of MOFs from the XPS Survey Analysis

	Co-TPA		Co-TPA/TA	
	binding energy (eV)	weight (%)	binding energy (eV)	weight (%)
Co 2p	781.33	36.26	781.41	34.51
O 1s	531.28	27.91	531.35	28.35
C 1s	284.33	35.83	284.37	37.14

According to Table 2, the cobalt content observed in Co-TPA decreased with the incorporation of C and O elements of TA into the matrix, and this result was compatible with the EDX results.

**Figure 7:**
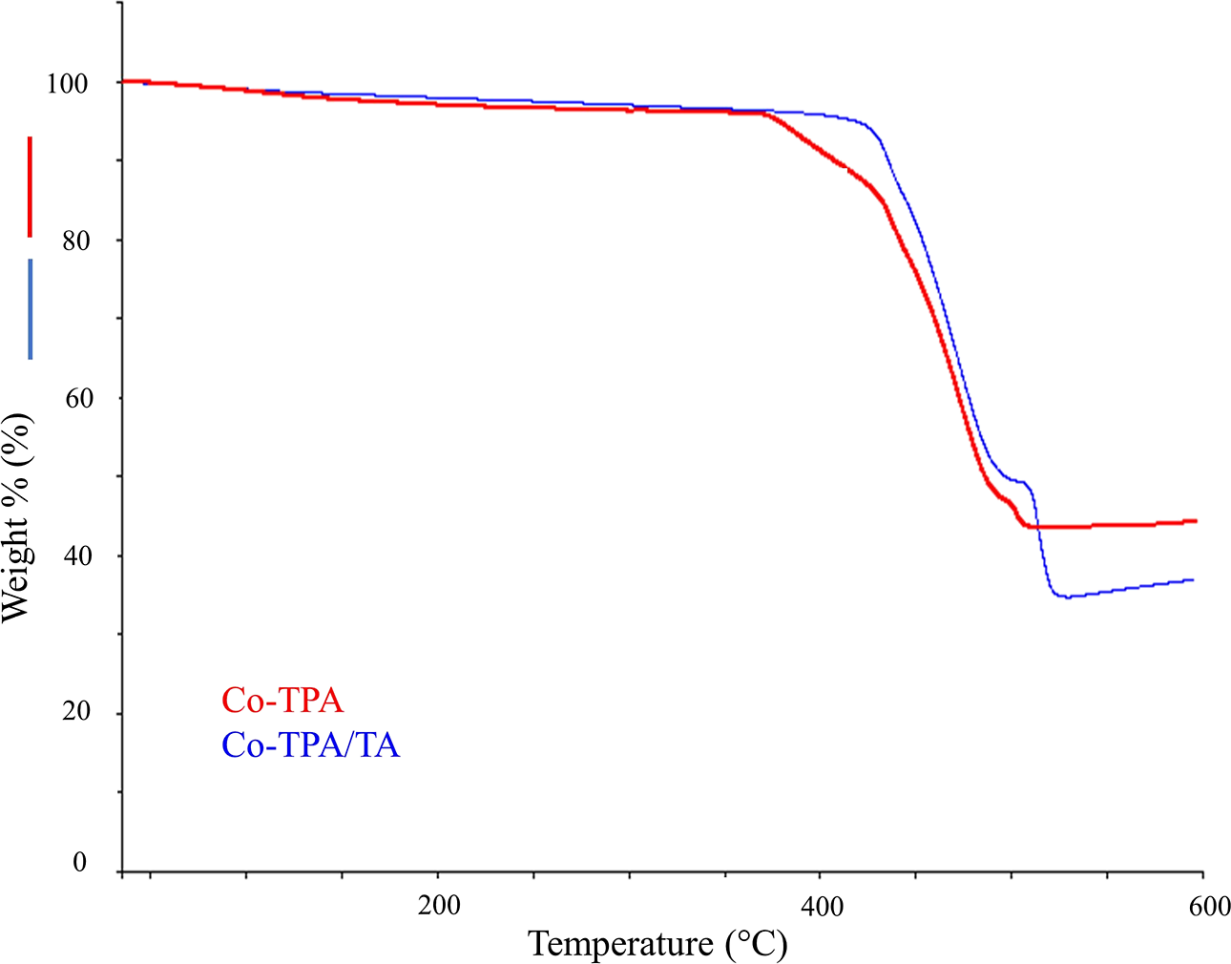
TG/DTG curves of Co-TPA and Co-TPA/TA.

The thermal behaviors of Co-TPA and Co-TPA/TA are given in Figure 7. The weight losses seen in the thermograms of the materials below 125 °C were due to the loss of moisture and water molecules coordinated to cobalt.^39^ A weight loss observed for the as-synthesized Co-TPA at 361 °C may have been due to the removal of residual DMF, which could potentially remain in the pores of the MOF during the synthesis procedure.^33,38^ Considering the dissociation of the metal–ligand skeleton, a sharp weight loss of 44.3% was observed at 473 °C for Co-TPA and no weight loss was seen as the temperature was raised further, indicating that TPA had fully broken down and only metallic oxide was left.^48^ In the thermogram of Co-TPA/TA, the second and the third stages of decomposition located at 475 and 514 °C with weight losses of 48.3 and 12.3%, respectively, were also attributed to the collapse of the framework. The overall weight loss observed for Co-TPA/TA was significantly higher than that of Co-TPA. It is estimated that the reason for this is the destruction of the organic structure of TA added to the matrix as well as the decomposition of the metal–ligand skeleton. The rises observed in the decomposition temperatures denoted that TA modification enhanced the thermal stability of Co-TPA/TA compared to Co-TPA.

### 3.2 Antimicrobial Activities of Co-TPA and Co-TPA/TA

**Figure 8:**
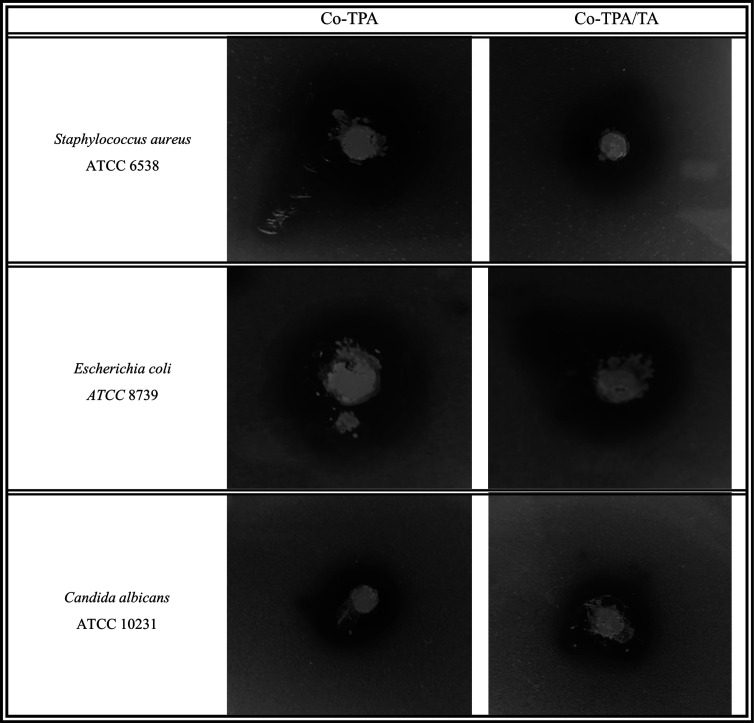
Antimicrobial activities of Co-TPA and Co-TPA/TA on *S. aureus*, *E. coli,* and *C. albicans*.

The National Committee for Clinical Laboratory Standards recommends the agar plate diffusion assay as the standard procedure based on the Bauer et al. method.^49^ Antimicrobial activities of Co-TPA and Co-TPA/TA were determined using agar diffusion assay, and the results were compared to those of standard antibiotic (amoxicillin). The inhibition zone values determined for Co-TPA, Co-TPA/TA, and standard antibiotics are shown in Figure 8 and Table 3. All cobalt-based MOFs exhibit potent antimicrobial activity, with an inhibitory diameter of roughly 20 mm compared with normal amoxicillin. All of the substances under investigation caused a 19 mm suppression of the yeast. This experiment demonstrates that MOFs can diffuse into the media, stopping the bacteria and yeast from growing. The ligand in a cobalt-based MOF that Zhuang et al.^50^ described as a disinfectant with high potency for inactivating *E. coli* was tetrakis(3,5-dicarboxyphenyl)-oxamethyl methane acid. The process by which metal ions are liberated from the crystal structures of MOFs and released into the surrounding physiological environment is known as metal ion release, and it is thought to be the primary source of the antimicrobial action of MOF, and the mechanism of release of metal ions and organic ligands and their synergistic impact has been thoroughly explored.^51^ Bacterial cells are destroyed as a result of the liberated metal ions’ ability to penetrate cell membranes.^52^

**Table 3:** Antimicrobial Activities of Co-TPA, Co-TPA/TA, and the Standard Antibiotic (Amoxycillin)^a^

antimicrobial/antifungal activities (inhibition zone, mm)			
material	S. aureus	E. coli	C. albicans
Co-TPA	20	21	19
Co-TPA/TA	14	20	19
A/30	14	16	-b

a A/30 amoxicillin, 30 μg.

b - not tested.

The broad-spectrum antimicrobial properties of several metal ions, including silver (Ag^+^), zinc (Zn^2+^), copper (Cu^2+^), iron (Fe^2+^ or Fe^3+^), lead (Pb^2+^), manganese (Mn^2+^), and cobalt (Co^2+^) ions, as well as their relative lack of toxicity to eukaryotic cells, have attracted research attention.^53−57^ MOFs’ chemical activity and stable, adjustable structure help regulate how they interact with bacterial compounds that are active,^58−61^ including biological activities. Numerous MOFs have undergone extensive research in the field of biomedicine due to their preferred antimicrobial activity, which is caused by a variety of unique physical and chemical characteristics (such as slow release of metal ions or organic substances and activity similar to an enzyme, photocatalytic, photothermal, or ultrasonic processes).

The superior antimicrobial activity of Co-TPA and Co-TPA/TA could generally be attributed to a number of characteristics, including the high surface area, distinctive shape and structure, porosity, their diffusion framework on the surface of microorganisms, and their impact on the bacterial cell’s surrounding environment.^62^ Additionally, when metal ions come into touch with the bacterial cell walls, the organic ligand in MOF serves as a reservoir for the metal ions and can interact with cations inside of the cell to produce oxygen-reactive species in the cytoplasm, which can break and alter DNA.^56,63,64^

In a study by Uflyand et al.,^40^ Co-MOF based on TPA and 1,10-phenanthroline was synthesized and the sorption and antioxidant and bactericidal properties were investigated. The phenanthroline ligand and intermolecular hydrogen bonds that were formed might have been the cause of the MOF’s strong antimicrobial action. As a result, the MOF’s cationic release property made it a perfect bactericidal agent for antimicrobial medications.^50,65−67^ Furthermore, the cobalt-ion-containing active centers of this MOF function as effective catalysts for lipid peroxidation of the plasmalemma’s bilipid layer, which ruptures the bacterial cell membrane and inactivates the bacterium. High and sustained antimicrobial activity is ensured by the nearly 100% catalyst recirculation used in the process.^68^

The antimicrobial activity of Co-TPA/TA depending on inhibition zone parameters was comparable to those in previous reports (Table 4). MOFs have concurrently shown notable long-term antimicrobial activities. The lengthy duration of action is attributed to the synergistic effect of the unique TA-modified cobalt-based MOF, which releases metal cations and organic ligands, explaining the excellent antimicrobial activity of the compound, especially against *E. coli* in this investigation.

**Table 4:** Comparison of Antimicrobial Activities of Co-MOF/TA against *E. coli*, *S. aureus*, and *C. albicans* with Previous Studies^a^

	antimicrobial activity (inhibition zone, mm)			
material	E. coli	S. aureus	C. albicans	references
BUC 51	13	-	-	(54)
[Ag_2_(O-IPA)(H_2_O)·(H_3_O)]	20	16	-	(55)
MCu/MOF	17	10	-	(69)
[Co(bimip)(H_2_O)0.5]·0.5H_2_O	-	12	11	(70)
Co-MOF	19.2	19.5	-	(71)
ZIF-67@silk (U)	11	16	-	(72)
Zn-MOF	-	13.5	13.3	(73)
ZIF-67@MIL-125-NH_2_	9	13	13	(74)
Co-TPA/TA	20	14	19	this study

a - Not tested.

## 4 Conclusions

This work presents a novel TA-modified cobalt-based MOF (Co-TPA/TA) successfully synthesized via a conventional and facile method. The morphology, structure, and composition of this MOF-based material were characterized with some spectroscopic methods and compared with the as-synthesized Co-TPA. Confirmation of the coordination of TPA ligand with metallic cobalt, the presence of Co within the studied matrix, and the crystalline nature of the MOFs were well-defined by FTIR, XRD, SEM-EDX, XPS, and ICP-OES analyses. TA modification seemed to be successfully achieved according to the obtained results. In comparison with the as-synthesized Co-TPA, the thermal stability was found to be slightly enhanced for Co-TPA/TA depending on the TA modification, and it was observed that the novel MOF-based material was thermally stable up to approximately 400 °C. Moreover, Co-TPA/TA was evaluated as an antimicrobial agent against different types of pathogens and showed antimicrobial activity against bacteria, *S. aureus* and *E. coli*, and yeast, *C. albicans*, with an inhibition zone ranging between 14 and 20 mm. The moderate antimicrobial activity against bacterial and yeast cultures was demonstrated by Co-TPA/TA. The potential antimicrobial pathways may be due to ATP level reduction, reactive oxygen species production, and membrane damage from cobalt ion release and the creation of an alkaline microenvironment. These findings indicated the promise of Co-TPA/TA as a high-potential antimicrobial material for usage in a range of biological and pharmaceutical applications.
